# Hematopoietic Stem Cell Transplantation for Neurological Disorders: A Focus on Inborn Errors of Metabolism

**DOI:** 10.3389/fncel.2022.895511

**Published:** 2022-05-26

**Authors:** Pedro de Vasconcelos, João F. Lacerda

**Affiliations:** ^1^Serviço de Hematologia e Transplantação de Medula, Hospital de Santa Maria, Centro Hospitalar Universitário Lisboa Norte, Lisbon, Portugal; ^2^JLacerda Lab, Hematology and Transplantation Immunology, Instituto de Medicina Molecular João Lobo Antunes, Faculdade de Medicina da Universidade de Lisboa, Lisbon, Portugal

**Keywords:** hematopoietic stem cell transplantation, hematopoietic stem cell gene therapy, neurological disorder, leukodystrophy, demyelination diseases, microglia, brain repair

## Abstract

Hematopoietic stem cells have been investigated and applied for the treatment of certain neurological disorders for a long time. Currently, their therapeutic potential is harnessed in autologous and allogeneic hematopoietic stem cell transplantation (HSCT). Autologous HSCT is helpful in immune-mediated neurological diseases such as Multiple Sclerosis. However, clinical benefits derive more from the immunosuppressive conditioning regimen than the interaction between stem cells and the nervous system. Mainly used for hematologic malignancies, allogeneic HSCT explores the therapeutic potential of donor-derived hematopoietic stem cells. In the neurological setting, it has proven to be most valuable in Inborn Errors of Metabolism, a large spectrum of multisystem disorders characterized by congenital deficiencies in enzymes involved in metabolic pathways. Inborn Errors of Metabolism such as X-linked Adrenoleukodystrophy present with brain accumulation of enzymatic substrates that result in progressive inflammatory demyelination. Allogeneic HSCT can halt ongoing inflammatory neural destruction by replacing hematopoietic-originated microglia with donor-derived myeloid precursors. Microglia, the only neural cells successfully transplanted thus far, are the most valuable source of central nervous system metabolic correction and play a significant role in the crosstalk between the brain and hematopoietic stem cells. After transplantation, engrafted donor-derived myeloid cells modulate the neural microenvironment by recapitulating microglial functions and enhancing repair mechanisms such as remyelination. In some disorders, additional benefits result from the donor hematopoietic stem cell secretome that cross-corrects neighboring neural cells *via* mannose-6-phosphatase paracrine pathways. The limitations of allogeneic HSCT in this setting relate to the slow turnover of microglia and complications such as graft-vs.-host disease. These restraints have accelerated the development of hematopoietic stem cell gene therapy, where autologous hematopoietic stem cells are collected, manipulated *ex vivo* to overexpress the missing enzyme, and infused back into the patient. With this cellular drug vehicle strategy, the brain is populated by improved cells and exposed to supraphysiological levels of the flawed protein, resulting in metabolic correction. This review focuses on the mechanisms of brain repair resulting from HSCT and gene therapy in Inborn Errors of Metabolism. A brief mention will also be made on immune-mediated nervous system diseases that are treated with this approach.

## Introduction

Hematopoietic stem cells (HSC) have historically been used to treat deadly blood disorders. The first report of allogeneic infusion of HSC in human malignancies dates to 1957 (Donnall Thomas et al., 1957) and since then, the field of HSC transplantation (HSCT) has evolved tremendously. In the modern era, HSCT is the only available stem cell therapy with demonstrated robust clinical benefits for the treatment of human diseases. Several non-hematologic conditions are currently treated with HSCT, including nervous system disorders. Autologous and allogeneic hematopoietic stem cell transplantation (AHSCT and Allo-HSCT, respectively) are successful approaches for the treatment of specific neurological conditions.

AHSCT is able to arrest neuroinflammation in diseases such as Multiple Sclerosis, Neuromyelitis Optica Spectrum Disorder and Myasthenia Gravis. However, clinical benefits result mainly from the peripheral immune reset induced by the conditioning regimen rather than the direct interaction between HSC and the nervous system (Sharrack et al., 2020). Despite the potential benefits of the graft-vs.-autoreactivity effect and self-tolerant immune reconstitution (van Wijmeersch et al., 2007), Allo-HSCT has been seldomly used in auto-immune neurological disorders due to a high risk of treatment-related mortality (Greco et al., 2019; Sharrack et al., 2020). Clinical indications of Allo-HSCT for the treatment of neurological conditions are mostly restricted to Inborn Errors of Metabolism (IEM), a large spectrum of congenital multisystemic diseases characterized by specific enzyme deficiencies involved in metabolic pathways. IEM presenting with progressive inflammatory demyelination or neurodegeneration such as Hurler Syndrome, Metachromatic Leukodystrophy, Krabbe’s Disease and X-Linked Adrenoleukodystrophy benefit from Allo-HSCT before high central nervous system (CNS) disease burden is present (Tan et al., 2019). Similar to other disease settings, the therapeutic success of HSCT in IEM has been experienced before clear biological underpinnings were elucidated. In this setting, Allo-HSCT has been shown to halt and even reverse CNS injury through two main mechanisms that occur after HSC migration to the brain parenchyma: (1) local replenishment of the lacking enzyme through cross-correction of neighboring neural cells and (2) restoration of defective microglia function by donor-derived myeloid cells (Ferrante et al., 1971; Krivit et al., 1995; Capotondo et al., 2012; Wolf et al., 2020).

The need for a compatible donor and complications such as graft-vs.-host disease are current limitations of Allo-HSCT. They have paved the way for the development and application of Hematopoietic Stem Cell Gene Therapy (HSC-GT) in IEM with neurological involvement. HSC-GT relies on the use of autologous HSC that are manipulated *ex vivo* to overexpress the missing enzyme through transduction with lentiviral vectors encoding the transgene of interest. Infusion of these manipulated HSC results in supraphysiological delivery of the lacking protein. In this manner, autologous HSC are used as cellular vehicles of the missing enzyme to affected tissues such as the brain, enabling metabolic correction, clearance of storage materials, and ultimately repair. Currently, HSC-GT is emerging as an alternative to Allo-HSCT in the treatment of certain IEM such as X-linked Adrenoleukodystrophy, Hurler Syndrome, and Metachromatic Leukodystrophy (Eichler et al., 2017; Gentner et al., 2021; Fumagalli et al., 2022).

In this review, we highlight the current clinical use of HSCT in the treatment of central nervous system diseases and the underlying therapeutic mechanisms. Firstly, the principles of HSCT are introduced, followed by a brief exploration of the role of HSCT in immune-mediated neurological diseases. Then we will focus on Inborn Errors of Metabolism with CNS involvement. Further details are presented on the clinical manifestations, neuropathology and therapeutic pathways in the specific cases of Hurler Syndrome, Metachromatic Leukodystrophy, Krabbe’s Disease, and X-linked Adrenoleukodystrophy. Finally, we summarize recent data on Hematopoietic Stem Cell Gene Therapy as a cellular drug vehicle to the brain. This review aims to detail brain repair mechanisms in neurological conditions treated with HSCT and highlight how current benefits and limitations of this approach can be used as a model for future developments in the field.

## Principles of Hematopoietic Stem Cell Transplantation

Autologous Hematopoietic Stem Cell Transplantation is a type of transplant procedure characterized by three steps: (1) mobilization and harvest of autologous HSC, (2) administration of a high-dose immunosuppressive conditioning regimen, and (3) infusion of HSC (Carreras et al., 2019). The clinical benefits of AHSCT derive exclusively from the immune-ablative properties of the conditioning regimen (Sharrack et al., 2020). Despite being administered with the intent to halt the inflammation driving the underlying disorder, high dose treatment with chemotherapy and other immunosuppressive agents results in a protracted bone marrow failure state. Collected HSC are then reinfused to rescue bone marrow hematopoietic recovery in the ablated patient (Carreras et al., 2019). Immune reconstitution is ideally re-conditioned toward self-tolerance, which is further beneficial if autoimmunity is present (Cencioni et al., 2022). Importantly, HSC only differentiate into hematopoietic cells in this context and do not seem to participate in tissue repair outside of the bone marrow compartment.

Allogeneic Hematopoietic Stem Cell Transplantation is a procedure characterized by the infusion of donor-derived HSC to recipients previously treated with high-intensity conditioning regimens. After high dose chemotherapy and immunosuppression, donor-derived HSC home to and engraft in the bone marrow of the recipient and ideally reconstitute as a new and healthy lymphohematopoietic system. In contrast to AHSCT, where the donor and recipient are the same, genetic disparities must be overcome to prevent graft rejection. Accordingly, the conditioning regimens used are more intensive than in AHSCT, which contributes to a more efficient disease eradication in the context of hematologic malignancies. Also, post-transplant immunosuppressive prophylaxis is mandatory to prevent potentially life-threatening donor-to-recipient alloreactivity—graft-vs.-host disease (GvHD). Toxicity and treatment-related mortality are much more significant than AHSCT, and careful patient and donor selection are of utmost importance (Carreras et al., 2019). This type of HSCT has been used since the 50s to treat deadly blood cancers such as acute leukemia (Donnall Thomas et al., 1957). Currently, the clinical indications of Allo-HSCT extend much beyond hematologic malignancies and certain nervous system conditions do benefit from this treatment modality.

## Hematopoietic Stem Cell Transplantation for Immune-Mediated Neurological Diseases

Immune-mediated Neurological Diseases comprise a vast group of auto-immune neuroinflammatory conditions characterized by loss of self-tolerance in the nervous system compartment. These conditions can result in devastating clinical consequences to affected patients and most treatment modalities are based on strategies to suppress or modulate the dysfunctional immune system. In this section, we will focus on the role of HSCT in Multiple Sclerosis and other immune-mediated neurological diseases.

### Autologous Hematopoietic Stem Cell Transplantation

In the context of neurological disorders, AHSCT is most frequently used for immune-mediated conditions. Multiple Sclerosis (MS), the most common chronic inflammatory CNS disease (Compston and Coles, 2002), was first reported to benefit from this treatment in 1997 (Fassas et al., 1997). Since then, and by 2021, 1738 patients with MS have been treated with AHSCT in Europe, representing the most common autoimmune indication for this type of HSCT (Snowden et al., 2021). According to the European Group for Blood and Bone Marrow Transplantation (EBMT) Guidelines, AHSCT should be offered to patients with highly active relapsing-remitting MS with high clinical and imaging inflammatory activity despite one or more lines of approved disease-modifying therapies (grade 1 recommendation). This approach has demonstrated acceptable toxicity and potentially permanent clinical benefits (Sharrack et al., 2020). Notably, the disease phase is crucial for maximum clinical benefit. Low neuroinflammatory activity usually renders disease unresponsive to AHSCT, as observed in secondary progressive MS (Ismail et al., 2019).

AHSCT is used in MS with the premise that high-dose chemotherapy and other immunosuppressive agents (the conditioning regimen) result in the depletion of autoreactive immunologic memory and subsequent self-tolerant immune regeneration—the so-called “immune reset.” In this setting, it has been shown that a shift toward tolerance in the T cell compartment occurs in post-AHSCT immune reconstitution, involving reduced Th1, Th17, and terminally differentiated effector memory cells and increased naïve T cells with a renewed TCR repertoire (Muraro et al., 2005; Cencioni et al., 2022). In fact, early post-AHSCT T-cell repertoire diversity correlates with improved clinical outcomes in MS (Muraro et al., 2005). Despite these observations, insufficient conditioning intensity, high degree of inflammation, and genetic predisposition may lead to persistent autoreactive immunologic memory, treatment failure and disease relapse.

AHSCT has also been used for intense immunomodulation of other neuroinflammatory conditions that have failed standard treatment, such as Neuromyelitis Optica Spectrum Disorder, Autoimmune Encephalitis, Stiff Person Syndrome, Myasthenia Gravis, Chronic Inflammatory Demyelinating Polyradiculoneuropathy, and systemic autoimmune diseases with neurological manifestations (Burman et al., 2018). Only a limited number of patients have been treated with this approach and mechanisms of action are extrapolated from the experience in MS. So far, clinical benefits seem modest and the EBMT states that refractory patients may be considered for AHSCT (grade 2 recommendation) (Sharrack et al., 2020).

### Allogeneic Hematopoietic Stem Cell Transplantation

The rationale for using Allo-HSCT in the treatment of immune-mediated neurological diseases resides in the concept that the auto-reactivity of the recipient may be more efficiently eliminated by higher intensity conditioning and as a result of the graft-vs.-autoimmunity effect (van Wijmeersch et al., 2007; Sharrack et al., 2020). However, these therapeutic benefits ensue at the expense of increased risk of sometimes prohibitive complications such as GvHD. Notably, secondary neuro-immune complications like the recently recognized encephalitides associated with neuronal cell-surface protein antibodies can develop due to aberrant immune reconstitution after Allo-HSCT. This may in turn, lead to a further increased management complexity (Pirotte et al., 2018; Nagai et al., 2019; Hümmert et al., 2021).

Allo-HSCT has been seldomly used in immune-mediated neurological disorders. Experience in MS is scarce and reported cases mainly consist of patients transplanted for concomitant hematologic malignancies (Mcallister et al., 1997; Mandalfino et al., 2000; La Nasa et al., 2004; Jeffery, 2007; Lu et al., 2009). A postmortem histopathological case-control study demonstrated that Allo-HSCT could not halt inflammatory CNS demyelination in four MS patients transplanted for various hematologic diseases (Lu et al., 2010). The authors attributed treatment failure to recipient immune cell persistence in the brain despite intensive immune ablation and amplification of CNS inflammation due to GvHD and opportunistic infections. In fact, acute GvHD has been shown to result in CNS inflammation associated with neuronal cytotoxicity due to intraparenchymal donor lymphocytic infiltration (Shortt et al., 2005; Saad et al., 2009) and increased numbers of TNF-producing activated microglia (Mathew et al., 2020). Therefore, given these observations, unless a fully matched sibling is used as a donor to reduce alloreactivity to a minimum, the risk of CNS GvHD and neurological deterioration may outweigh the potential benefit of this treatment modality in MS.

Other immune-mediated neurological diseases have been treated with Allo-HSCT, mainly in the context of refractoriness to AHSCT. Neuromyelitis Optica Spectrum Disorder seems to particularly benefit from this treatment approach as reported cases demonstrated long-term auto-antibody elimination and disease stabilization. Interestingly, clinical outcomes seem superior to AHSCT in this disease (Greco et al., 2014, 2019; Ceglie et al., 2019; Hau et al., 2020), which warrants further investigation.

The EBMT considers Allo-HSCT a developmental indication for immune-mediated neurological diseases (grade 3 recommendation) and ideally should be performed within the auspices of a prospective clinical trial (Sharrack et al., 2020).

The pro-inflammatory skewed and aberrant auto-immune-like responses sometimes observed after Allo-HSCT immune reconstitution may be an obstacle too significant to overcome for most neuroinflammatory conditions. In the future, harnessing endogenous protective pathways, namely polarizing microglia to the anti-inflammatory and tissue repairing M2 phenotype, may be explored to shield the CNS from pathologic systemic immune responses.

## Inborn Errors of Metabolism With Neurological Involvement

Inborn Errors of Metabolism comprise a heterogeneous spectrum of rare congenital multisystem disorders characterized by defects in genes encoding enzymes involved in intracellular metabolic pathways. IEM may not appear at first the obvious paradigm of CNS disorders since the affected genes are expressed ubiquitously. However, specific brain components such as myelin sheaths and the resident phagocytic cells microglia are substantially impaired by the accumulation of improperly degraded substrates. In this setting, the ability of HSC to migrate to the CNS and modulate its cellular composition and enzymatic secretome have been explored as therapeutic “Trojan Horses” or cellular drug vehicles. Allo-HSCT has been used to treat patients with rapidly progressive hereditary leukodystrophies for approximately three decades (Page et al., 2019). Recently, breakthroughs have been made with Hematopoietic Stem Cell Gene Therapy, with encouraging results compared to classic HSCT approaches in this setting (Biffi, 2017).

This section will focus on microglia as the primary neural cell driving neurological disease phenotype in IEM and its role as a therapeutic target and model for future treatment approaches. An overview of all IEM is beyond the scope of this review, and particular emphasis will be made on IEM that benefit from HSCT as a treatment modality, such as certain Lysosomal Storage Diseases and the Peroxisomal Disorder X-Linked Adrenoleukodystrophy. A summary of the events resulting in brain repair with Allo-HSCT in IEM with neurological involvement is presented in Table 1.

**TABLE 1:** Events that result in brain repair with allogeneic hematopoietic stem cell transplantation in Inborn Errors of Metabolism with neurological involvement.

1. *Brain-conditioning:* Busulfan-based conditioning regimen leads to depletion of malfunctioning host microglia, creating a permissive niche inside the brain microenvironment.
2. *Brain engraftment*: HSC and myeloid progenitors cross the BBB and occupy the microglial vacant niche, where they differentiate into microglia/macrophage.
3. *Restored microglial function*: donor-derived microglia/macrophages recapitulate native microglia function resulting in: • Improved scavenging and immune surveillance • Improved myelin debris clearance • Increased pro-regenerative M2 phenotype microglia • Differentiation of oligodendrocytes precursors into myelin-producing cells • Oligodendrocyte survival and proliferation signaling • BBB dysfunction correction
4. *Cross-correction*: donor-derived microglia/macrophages secrete functional enzyme to the extracellular matrix, which is then captured by neighboring oligodendrocytes and astrocytes through paracrine 6MP pathways.

*HSC, hematopoietic stem cells; BBB, blood-brain barrier; 6MP, 6-mannose-phosphate.*

### Microglia: A Cellular Intermediate of the Hematopoietic and Central Nervous Systems

Microglia are the only CNS-resident innate immune cells, comprising roughly 10% of the total glial population. In contrast to their neighboring glial counterparts, microglia are of hematopoietic myeloid origins and share ontogeny with peripheral blood monocytes and tissue macrophages (Ginhoux et al., 2010). As a subtype of phagocytic tissue cells, microglia play critical roles in debris scavenging and immune surveillance through cytokine signaling and Fc and complement receptor phagocytic pathways (Prinz and Mildner, 2011). Resting microglia constantly scan the brain parenchyma for tissue damage and foreign cells through extension and retraction of their highly motile ramified processes (Nimmerjahn et al., 2005). Apart from their role in innate immunity, they serve as fundamental effectors of brain homeostasis, participating in several events such as control of the proliferation and differentiation of oligodendrocytes upon injury; formation of synaptic connections; pruning of synapses and myelin sheaths; and clearance of apoptotic neurons and myelin debris after injury (Paolicelli et al., 2011; Waisman et al., 2015; Cignarella et al., 2020; Hughes and Appel, 2020). Microglia seem to interact with virtually all brain cells and form cellular grids within the CNS that are maintained throughout the lifespan of the organism.

The origin of microglia during development has been a matter of intense debate for decades. The currently accepted view is that they originate in yolk-sac macrophages that colonize embryonic tissues such as the developing brain, where they proliferate *in situ* and are maintained throughout adulthood by a local progenitor pool (Ginhoux et al., 2010). Of note, at this developmental stage, myeloid cell generation in the fetal liver has not started, highlighting the independent origin of microglia compared to classical macrophages that derive from monocytes (Naito et al., 1990; McGrath et al., 2003). In contrast to all other tissue macrophages, fetal monocytes do not substitute the microglial progenitor pool during development because the blood-brain barrier (BBB) is formed during this embryonic phase and prevents monocyte migration to the brain (Daneman et al., 2010). Using parabiotic models where the circulatory systems of mice were surgically connected while keeping organs separated, Ajami et al. (2007) observed no brain influx of monocytes or bone marrow cells despite the substantial mixture of circulating leucocytes. These results suggest that microglia maintenance depends exclusively on the self-renewal capacities of the microglia progenitor pool residing in the brain (Ajami et al., 2007). Given the relative isolation and apparent independence of microglia from peripheral blood replenishment, intrinsic defects in these cells result in profound brain cell injury that can accumulate significantly over time.

Despite these observations, under non-physiological conditions explored in animal models of Allo-HSCT, such as irradiation-induced BBB dysfunction, monocytes, and bone marrow cells can enter the brain parenchyma and differentiate into microglia-like cells (Mildner et al., 2007). Besides BBB dysfunction, other mechanisms seem to induce the recruitment of these cells to the CNS. Specific chemotherapy agents used in conditioning regimens before Allo-HSCT may result in the so-called “brain conditioning,” which creates a permissive niche for brain engraftment of donor-derived myeloid cells. Sailor et al. (2022) have recently demonstrated that Busulfan, an alkylayting agent commonly used in conditioning regimens, causes host microglial senescence, cell cycle arrest and regenerative capacity exhaustion. This, in turn, results in more efficient trafficking of donor-derived macrophages to the brain that successfully engraft and become resident. The authors propose that when microglial cell loss reaches a critical density, the brain niche becomes permissive for donor cell engraftment (Sailor et al., 2022). Capotondo et al. (2012) also reported on Busulfan, showing that it depletes functionally defined microglia precursors in the brain, allowing increased turnover by donor-derived myeloid cells *via* CCR2 signaling. In this study, microglia replacement seemed to result primarily from bone marrow-derived microglia precursors rather than from the progeny of donor hematopoietic stem cells engrafted in the bone marrow niche. This observation led the authors to propose that post-Allo-HSCT microglia reconstitution may occur independently from hematopoietic engraftment in the bone marrow compartment. In summary, transplant models, which do not reflect normal physiology, have demonstrated that, under the appropriate conditions, microglia can efficiently be replaced by allogeneic adult bone marrow cells, which become an integral part of the CNS cellular network.

In order to replenish the vacant microglial niche, it may be possible that induced depletion of these cells creates a chemokine gradient capable of recruiting bone-marrow precursors and peripheral blood monocytes to the brain through a transiently permeable BBB.

### Lysosomal Storage Diseases

Lysosome Storage Diseases (LSD) are a vast group of congenital conditions characterized by deficiencies in specific proteins involved in lysosomal pathways such as soluble lysosomal hydrolases, membrane proteins, and transporters. The lysosome is an intracellular organelle responsible for numerous functions, including degradation and recycling of intra and extracellular material, cellular membrane repair, lipid, and metabolite exchange between organelles and energy metabolism regulation (Reddy et al., 2001; Medina and Ballabio, 2015; Todkar et al., 2017). Defects in these functions result in impaired catabolism of substrates, leading to accumulation initially in the endosomes and lysosomes and subsequently in other intracellular compartments and the extracellular space. Ultimately these processes culminate in cell death and tissue dysfunction (Campos and Monaga, 2012; Biffi, 2017). The phagocytic system is particularly vulnerable in these conditions because monocytes/macrophages are of high metabolic demand and rely heavily on efficient lysosomal machinery for proper scavenging.

LSD have highly variable clinical manifestations that are determined by the tissue distribution of substrate accumulation. Roughly 70% of affected patients present with CNS involvement, making LSD the most common cause of neurodegeneration in the pediatric setting (Cartier et al., 2014; Biffi, 2017).

Microglia dysfunction and substrate accumulation in other neural cells and structures are the main mechanisms of brain injury in LSD. Deficient microglia amplify destructive pathways in the brain microenvironment due to impaired modulation of injury responses. Additionally, a skew in the immune phenotype characterized by pro-inflammatory and non-regenerative signaling develops as a secondary event. All these combined events result in demyelination and ineffective remyelination that define the neuropathology of most of these disorders (Suzuki, 1998).

CNS disease is one of the critical factors affecting the long-term survival of LSD patients, and effective treatment approaches heavily rely on the capacity to alleviate brain injury. For this purpose, treatment with Allo-HSCT has shown considerably better neurological outcomes compared to other interventions such as Enzyme Replacement Therapy (ERT) (Enns and Huhn, 2008).

### Enzymatic Cross-Correction: The Main Therapeutic Principle of Allo-HSCT in Lysosome Storage Diseases

In normal cells, newly synthesized lysosomal enzymes require the addition of mannose-6-phosphate (M6P) residues in the Golgi apparatus for correct trafficking to the lysosome as proenzymes. However, almost half of the M6P tagged proenzymes can escape the lysosome and are secreted out to the extracellular space, where they can be recaptured by neighboring cells harboring M6P membrane receptors. After internalization, these proenzymes can ultimately reach the lysosome of adjacent cells (Cartier et al., 2014; Biffi, 2017).

Cross-correction refers to the process of functional protein transfer from a healthy cell into adjacent deficient cells (Harrison et al., 2013). In the setting of lysosomal proteins, this secretion-recapture system is based on the M6P paracrine pathway. The proof-of-concept of cross-correction in LSD was demonstrated in 1968 by Fratantoni et al. (1968). Cultured skin fibroblasts from Hurler and Hunter Syndrome (Mucopolysaccharidosis type I and II, respectively) patients showed correction of the biochemical defect when the cells of the two genotypes were mixed with each other or with healthy cells. A corrective factor was postulated to be released to the medium and correct the defect of neighboring cells after internalization (Fratantoni et al., 1968). It is currently acknowledged that these events occur through 6MP pathways and that the corrective factor is a functional enzyme secreted by wild-type cells.

Based on these observations, ERT was developed as an obvious approach to cross-correction. Despite its long success story in various LSD, ERT cannot correct tissues difficult to access, such as the brain. Delivered enzymes do not cross the BBB, and this approach fails to correct CNS lysosomal metabolism (Tokic et al., 2007; Enns and Huhn, 2008). Additionally, ERT depends on long-term use, which can result in the development of anti-enzyme antibodies rendering treatment ineffective (Tokic et al., 2007).

To circumvent the inherent limitations of ERT, Allo-HSCT was pursued as a one-time intervention capable of providing enzyme-producing cells to several tissues, including the CNS (Enns and Huhn, 2008). The therapeutic role of Allo-HSCT in LSD relies mainly on cross-correction (Tan et al., 2019). In this procedure, metabolic competent donor-derived myeloid precursors are transplanted, migrate to tissue, and differentiate as macrophages (Capotondo et al., 2012). Then, the secretome of macrophages can correct the enzymatic deficiencies of neighboring cells through the M6P paracrine pathway (Biffi, 2017). Of note, the transplanted cells derive from HSC capable of self-renewing, therefore representing a continuous endogenous source of the missing enzyme. In the brain parenchyma, this process is achieved through the action of microglia, the most valuable source of CNS metabolic correction (Capotondo et al., 2012). Donor-derived macrophages/microglia express functional enzymes and can cross-correct neighboring neural cells that lack the critical protein, such as oligodendrocytes and astrocytes (Biffi, 2017).

The ability of Allo-HSCT to cross-correct brain tissue represents the central rationale for using this treatment approach in most LSD (Krivit et al., 1995). However, as explained next, certain LSD and the Peroxisomal Disorder X-Linked Adrenoleukodystrophy seem to benefit from additional mechanisms resulting from microglia replacement not directly related to enzyme replenishment. It is becoming increasingly apparent that restoration of some of the functions of these CNS myeloid cells, such as scavenging storage material and pro-neurogenic modulation of the brain microenvironment, also play critical therapeutic roles (Plemel et al., 2013; Lloyd et al., 2019).

### Lysosome Storage Diseases That Benefit From Allo-HSCT

#### Hurler Syndrome

Hurler Syndrome, the most severe phenotype of Mucopolysaccharidosis Type I (MPS IH), is characterized by loss of function variants of the IDUA gene, which encodes alpha-L-iduronidase. The lack of this enzyme results in ineffective catabolism of glycosaminoglycans that accumulate in multiple tissues, including the CNS (Muenzer, 2011). The pathophysiology of neurodegeneration in patients with MPS IH is poorly understood, but neural dysfunction from secondary glycosaminoglycan-induced ganglioside accumulation is thought to play an important role (Walkley, 2004). Defective cerebrospinal fluid reabsorption due to glycosaminoglycan accumulation in the meninges and arachnoid villi may result in communicating hydrocephalus, which can contribute to neurocognitive deterioration (Matheus et al., 2004). Ultimately, progressive cerebral atrophy develops and patients commonly present with an early-onset rapidly progressive disease with neurocognitive regression and death within the first decade of life (Muenzer et al., 2009).

MPS IH is the paradigm disorder of Allo-HSCT in IEM. The first report of HSCT in this disease dates to 1981 when a 1-year-old boy, after failing to engraft with HSC from his father, achieved clinical improvement with normalized enzyme plasma levels after successful engraftment with cells from his mother (Hobbs et al., 1981). It is worth mentioning that the ontogeny of microglia had not yet been deciphered in the 80s. The decision to pursue this treatment approach was based on the acknowledgment that donor-derived monocytes represented a valuable source of systemic metabolic correction.

Currently, Allo-HSCT with pretransplant or peritransplant ERT is a considered standard treatment for MPS IH (de Ru et al., 2011). As previously mentioned, the therapeutic benefits result from donor-derived myeloid cells that differentiate into tissue macrophages and cross-correct neighboring defective cells *via* M6P-dependent paracrine pathways (Fratantoni et al., 1968; Tan et al., 2019). In contrast to ERT, Allo-HSCT has the advantage of improving neurocognitive decline in affected patients (Krivit et al., 1995; Aldenhoven et al., 2008, 2015). The EBMT recommends this treatment modality for patients up to 30 months of age with no or minimal cognitive impairment and states that late transplantation will probably not meaningfully alter the natural history of the disease (Carreras et al., 2019). Underlining the importance of early Allo-HSCT, brain engraftment of donor-myeloid cells is a slow process that can take up to 1 year, which may lag behind neurological disease progression. This explains why certain patients have slow improvement or even CNS deterioration after HSCT (Boelens and van Hasselt, 2016; Biffi, 2017). In this setting, matched siblings should not be used as donors if they are disease-allele carriers and umbilical cord blood is currently considered the preferred graft source (Prasad and Kurtzberg, 2010; Carreras et al., 2019). Notably, newborn screening programs are expected to improve clinical outcomes in patients affected by MPS IH due to shorter diagnosis-to-transplantation time (Taylor et al., 2019).

#### Metachromatic Leukodystrophy

Metachromatic Leukodystrophy (MLD) is characterized by mutations in the ARSA gene, leading to deficiency of arylsulfatase and accumulation of sulfatides that are enriched in myelin sheaths. Sulfatide accumulation results in demyelination and inhibits oligodendrocyte precursor differentiation into myelin-producing cells, preventing remyelination. Therefore, patients affected with MLD present progressive inflammatory CNS and peripheral nervous system demyelination (Givogri et al., 2008; van Rappard et al., 2015). Of note, in an autopsy study, Bergner et al. (2019) demonstrated that severe dysfunction and microglia death precedes the decay of oligodendrocytes and reduction of myelin density. The authors hypothesized that oligodendrocytes initially outsource myelin degradation to metabolically incompetent microglia to protect the surrounding tissue, leading to toxic material sequestration. Although beneficial at first, this mechanism leads to progressive microglia dysfunction and secondary severe tissue injury (Bergner et al., 2019). In this manner, phagocytic lysosomal dysfunction seems to represent one of the major drivers of brain tissue injury in MLD.

Three MLD subtypes are defined depending on age at presentation: late-infantile, up to 30 months of age; juvenile, 30 months to 15 years of age; adult, over 15 years of age. Late-infantile MLD is the most common and severe phenotype, and patients invariably die in 2–3 years due to rapidly progressive neurological decline (Biffi et al., 2008). The very rapid pace of this phenotype means that diagnosis is usually made when severe CNS involvement is present, which renders Allo-HSCT ineffective in preventing progression (Wynn, 2011; Carreras et al., 2019). The window of opportunity of HSCT is probably too narrow for patients with late-infantile MLD unless prenatal *in utero* diagnosis is made. In contrast, the attenuated and later forms of the disease (juvenile and adult MLD) do benefit from early Allo-HSCT before significant symptomology is present (Boucher et al., 2015; Groeschel et al., 2016; van Rappard et al., 2016). Similar to the experience of HSCT in MPS IH, the replacement of microglia by donor-derived progeny is a slow process compared to the rapid disease progression, which may result in transplant failure (Tan et al., 2019).

In contrast to MPS IH, clinical benefits with HSCT in MLD seem to derive minimally from cross-correction. *In vitro* studies suggest that arylsulfatase secreted by microglia lacks 6MP markers, leading to the inability of neighboring cells to endocytose the enzyme through this pathway (Muschol et al., 2002). In an autopsy study of MLD patients treated with Allo-HSCT, Wolf et al. (2020) demonstrated that arylsulfatase was present in donor monocytes/macrophages but not on neighboring cells such as oligodendrocytes and astrocytes. Notwithstanding, increased numbers of oligodendrocyte precursors and mature myelin-forming oligodendrocytes were noted in transplanted patients, suggesting that Allo-HSCT supports the survival, proliferation and differentiation of these glial cells (Wolf et al., 2020). This supportive effect most likely results from indirect effects of metabolically competent donor-derived macrophages/microglia that clear extracellular sulfatides, efficiently scavenge brain microenvironment, and digest potent inhibitors of oligodendrocyte precursor maturation such as myelin debris (Plemel et al., 2013; Lloyd et al., 2019). Also, in this study, the presence of intermediately activated microglia was associated with evidence of remyelination in the white matter of transplanted patients, which argues for the supportive role of M2 phenotype microglia. This observation aligns with previously known macrophage polarization dynamics during myelin repair. After myelin loss, the timely polarization of these cells from a pro-inflammatory (classically activated, M1) into a pro-regenerative phenotype (alternatively activated, M2) results in the maturation of oligodendrocyte progenitors into myelin-producing cells (Franklin and Ffrench-Constant, 2008; Miron et al., 2013). Considering all this data, the benefits of Allo-HSCT in MLD seem to be mostly related to the intense immunomodulation and improved myelin metabolism homeostasis that comes from the brain engraftment of metabolically fit donor-derived cells. Cross-correction, if present at all in this setting, probably contributes only residually to brain repair mechanisms.

#### Krabbe’s Disease

Krabbe’s Disease (KD), also known as Globoid Cell Leukodystrophy, is characterized by mutations in the GALC gene that encodes the lysosomal enzyme galactocerebrosidase. Low or absent enzyme activity leads to the accumulation of galactolipids such as psychosine and consequent central and peripheral nervous system inflammatory demyelination (Page et al., 2019). The presence of numerous macrophages containing undigested galactolipids (globoid cells), astrocyte gliosis and almost total loss of myelin and oligodendrocytes represent the pathologic hallmarks of the disease (Wenger et al., 2019). Patients affected by KD present with symptoms either before or after 6 months of age, defining early-infantile and late-infantile KD, respectively. Early-infantile KD, the most common phenotype, is a rapidly progressive disease and patients usually die before the age of 3 due to neurological deterioration (Duffner et al., 2011). In line with other IEM, Allo-HSCT does not offer a clinical benefit after symptom onset as it cannot reverse demyelination after it has begun (Escolar et al., 2005). If diagnosed early, either *in utero* or at birth, affected asymptomatic patients can be considered for Allo-HSCT (Page et al., 2019). Due to the exceedingly early onset of disease, newborn screening is particularly relevant in early-infantile KD. Allo-HSCT performed during the first 4 weeks of life correlates with higher overall survival and functionality (Wright et al., 2017). Data regarding HSCT in late-infantile KD is scarce, and the decision to proceed to Allo-HSCT should be individualized. The mechanism of action of Allo-HSCT in KD relies heavily on cross-correction of neighboring neural cells by donor-derived HSC progeny. However, as described previously, the natural history of the disease may outpace timely brain engraftment of donor cells resulting in treatment failure (Hoogerbrugge et al., 1988; Krivit et al., 1998).

#### Peroxisomal Disorders

Peroxisomal disorders are a vast group of congenital conditions characterized by defects in genes encoding peroxisomal matrix enzymes, membrane transporters or proteins required for the organelle biogenesis. Peroxisomes are intracellular organelles essential for multiple anabolic and catabolic reactions such as biosynthesis of phospholipids and bile acids, cellular redox metabolism and oxidation of fatty acids such as very long-chain fatty acids (Waterham et al., 2016). In this subsection we will solely focus on the most frequent Peroxisomal Disorder—X-Linked Adrenoleukodystrophy.

#### X-Linked Adrenoleukodystrophy

X-Linked Adrenoleukodystrophy (ALD) is characterized by mutations in the ABCD1 gene that encodes adrenoleukodystrophy protein, a membrane transporter of substrates from the cytosol to the peroxisome. As a consequence of absent adrenoleukodystrophy protein, there is defective transportation of very long-chain fatty acids to the peroxisome for oxidative degradation, resulting in CNS and adrenal tissue accumulation (Moser, 1997). Four major presentations of ALD have been defined: asymptomatic, adrenal failure, adrenomyeloneuropathy and cerebral inflammatory disease. Occurring in 40% of patients during childhood or adolescence, Cerebral ALD (CALD) is the most severe disease phenotype and is associated with progressive neurological impairment and early mortality if left untreated (Moser et al., 1992; Tran et al., 2017). Affected boys develop normally until 4–12 years of age, then cerebral demyelination starts and progresses slowly over 1–3 years with mild symptomatology. After this initial period, a devastating clinical course begins. Rapid cerebral inflammatory demyelination ensues, BBB is disrupted, and peripheral innate immune cells migrate to the active edges of demyelinating lesions. Within 2–4 years of the first symptoms, affected patients enter a vegetative state associated with a dismal prognosis (Eichler et al., 2008).

The pathogenesis of CALD is defined initially by two events triggered by selective accumulation of substrate in specific brain structures: (1) destabilization of myelin sheaths and demyelination; (2) direct dysregulation of oxidative phosphorylation and mitochondrial function of neuroaxons. These processes are followed by a rapidly progressive inflammatory demyelination state characterized by invasion of myelin debris-laden macrophages and BBB dysfunction (Eichler et al., 2008; Lund et al., 2012; López-Erauskin et al., 2013; Weinhofer et al., 2018). Microglial activation, apoptosis and impaired switching to M2 phenotype after myelin uptake additionally contribute to the pathogenesis (Cartier et al., 2014). Poor plasticity and pro-inflammatory skewing of microglia were demonstrated by Weinhofer et al. (2018). The authors showed that relentless myelin loss perpetuated because microglia response did not transit from destructive pro-inflammation into remodeling/regeneration after myelin uptake. This observation explains why, in contrast to other demyelinating disorders such as MS, there is a lack of spontaneous halt of inflammation in most CALD lesions (Weinhofer et al., 2018). Additionally, Bergner et al. (2019) reported that a severe reduction in microglia density precedes full-blown oligodendrocyte and myelin degeneration. This suggests that, as part of an initial defense mechanism, undegradable toxic lipids are shed from myelinating cells onto the local phagocytic system (outsourcing of myelin turnover to microglia). Therefore, similar to MLD, it seems that microglial injury constitutes the earliest and one of the main pathogenic events of the disease (Bergner et al., 2019). Defective microglia neuroprotection results in oligodendrocyte and axon loss which is further amplified by the inflammatory wave of the disease (Glezer et al., 2007). Additionally, intrinsic defects in monocytes, the most severely metabolically affected immune cells, contribute to the pathogenesis through an altered cytokine secretome that results in pro-inflammatory T-cell responses (Weber et al., 2014). The mentioned mechanisms result in the pathologic hallmark CALD, consisting of myelin and axon loss, perivascular infiltration of macrophages and lymphocytes, migration of reactive microglia to demyelinating lesions, loss of oligodendrocytes, and reactive astrocytosis (Ferrer et al., 2010).

Driven by the success in MPS IH, the first successful use of Allo-HSCT in childhood-onset CALD was reported in the 90s. Despite initially experiencing progression of the brain lesions, the affected boy completely reversed cerebral demyelination 18 months after transplantation and currently, at the age of 30, lives a normal life (Aubourg et al., 1990). Since then, long-term follow-up has confirmed that HSCT can arrest neuroinflammation in CALD, provided it is performed at an early stage when there still are limited brain lesions and absent to minimal neurological deficits (Cartier and Aubourg, 2010). After HSCT, demyelination usually progresses during the first 12–18 months before disease arrest, reflecting the pace of microglia replacement and of the ongoing natural course of disease (Kennedy and Abkowitz, 1997). Importantly, Allo-HSCT is contraindicated in patients with advanced CALD because it accelerates inflammatory demyelination and neurological deterioration (Cartier and Aubourg, 2010). Notwithstanding, after MPS IH, CALD is the second most common IEM treated with Allo-HSCT (Wynn, 2011).

The therapeutic principle of Allo-HSCT in CALD relies on the replacement of defective microglia by long-lasting metabolically fit donor-derived myeloid cells (Cartier and Aubourg, 2010). It has been postulated that brain engrafted donor-derived monocytes can assist in attenuating neuroinflammation and possibility correct existing BBB dysfunction through differentiation into non-inflammatory and alternatively activated M2 microglia (Orchard et al., 2019). Microglial turnover is believed to be induced by certain drugs used in the conditioning regimen, such as Busulfan, which was shown to modulate the brain microenvironment and “create space” for neuro-engraftment (Capotondo et al., 2012). After conditioning, donor-derived macrophage precursors infiltrate the brain and occupy the CNS myeloid niche usually inhabited by microglia, where they efficiently recapitulate their brain counterpart functions (Varvel et al., 2012). Of note, the replacement of microglia seems to result directly from donor HSC and not from the long-term progeny of cells engrafted in the bone marrow compartment (Ajami et al., 2007; Biffi, 2012). This observation aligns with recent reports revealing that low chimerism levels may be sufficient for disease arrest (Ikeda et al., 2022). Therefore, peripheral blood and bone marrow chimerism studies likely underappreciate the “CNS chimerism” levels of donor-derived microglia.

In contrast to MPS IH and KD, cross-correction does not contribute to brain repair in CALD because peroxisomal membrane proteins are not secreted to the extracellular matrix. Therefore, the successful restoration of microglial functions by donor-derived cells represents the major mechanism of action of HSCT in this disorder.

A summary of the pathogenesis of IEM presented in this review and the benefits of Allo-HSCT are provided in Table 2.

**TABLE 2:** Summary of the genetic defects, neuro-pathogenesis and benefits of Allo-HSCT in IEM with neurological involvement.

Disease	Genetic defect	Neuro-pathogenesis	Benefits of Allo-HSCT
HS	Loss of function variants of the IDUA gene (encoding alpha-L-iduronidase)	Glycosaminoglycan accumulation in meninges and arachnoid vili Ganglioside accumulation	Donor microglia induced cross-correction
MLD	Mutations in the ARSA gene (encoding arylsulfatase)	Accumulation of sulfatides in myelin sheaths leading to demyelination Inhibition of OP differentiation into myelin-producing cells Microglia dysfunction	Restored microglial dysfunction by donor-derived myeloid cells Clearance of myelin debris, digestion of extracellular sulfatides and induction of OP differentiation
KD	Mutations in the GALC gene (encoding galactocerebrosidase)	Accumulation of galactolipids in the central and peripheral nervous leading to demyelination	Donor microglia induced cross-correction
X-ALD	Mutation in the ABCD1 gene (encoding adrenoleukodystrophy protein)	Accumulation of VLCFA in myelin sheaths leading to demyelination Dysregulated oxidative phosphorylation and mitochondrial dysfunction in neuroaxons Microglial dysfunction Peripheral inflammatory response	Restored microglial dysfunction by donor-derived myeloid cells Pro-regenerative M2 phenotype, attenuation of neuro-inflammation, and improved phagocytic function

*HS, Hurler Syndrome; MLD, Metachromatic Leukodystrophy; KD, Krabbe’s Disease; X-ALD, X-Linked Adrenoleukodystrophy; OP, oligodendrocyte precursor; VLCFA, very long chain fatty acids.*

## Hematopoietic Stem Cell Gene Therapy

The development of HSC-GT in IEM with CNS involvement emerged from some of the limitations of Allo-HSCT observed over the years. Firstly, as previously mentioned, replacement of defective microglia by transplanted donor-derived myeloid cells is a slow process compared to the natural evolution of the disease (Hoogerbrugge et al., 1988; Kennedy and Abkowitz, 1997; Krivit et al., 1998; Boelens and van Hasselt, 2016; Biffi, 2017). In most cases, microglia turnover in the brain occurs not earlier than 1 year after transplant, which has resulted in narrower clinical indications for Allo-HSCT than initially expected. Symptomatic children with non-early disease cannot wait 1 year until demyelination arrest and deteriorate faster with Allo-HSCT, rendering them ineligible for the procedure (Carreras et al., 2019). Secondly, transplant requires a compatible donor because genetic disparities increase the risk of rejection and GvHD—a form of alloreactivity that helps to eliminate residual disease in hematologic malignancies, but that has no benefit in benign conditions. In fact, acute GvHD is a potentially fatal complication and chronic GvHD is associated with considerable long-term morbidity (Carreras et al., 2019). For these reasons, matched siblings are the preferred donors for benign conditions. However, in the setting of congenital disorders such as LSD and ALD, using a sibling donor carries the risk of transplanting cells with disease-causing alleles, resulting in the delivery of enzymatically deficient HSC (Biffi, 2017). Using matched unrelated donors bypasses this issue but depends on availability and it is a logistically cumbersome process that may result in treatment delay. Haploidentical family donors are quicker alternatives, but their use is associated with a higher risk of certain complications such as GvHD. For these reasons, umbilical cord blood has been used as an alternative to related or unrelated donors. However, slow hematopoietic engraftment with subsequent increased risk of infection and graft failure are observed with this graft source (Carreras et al., 2019). Finally, Allo-HSCT is associated with significant toxicities secondary to the conditioning regimen and GvHD management. Of note, Busulfan (a frequent component of the conditioning regimen) and calcineurin inhibitors (the backbone of GvHD prophylaxis) are well recognized neurotoxic agents (Bechstein, 2000; Ciurea and Andersson, 2009) that may result in devastating consequences in patients with baseline impairment of brain functional reserve.

Many of these limitations can be overcome with the use of HSC-GT. Similar to AHSCT, this procedure relies on the infusion of autologous HSC into a patient previously exposed to a conditioning regimen. However, in HSC-GT, the collected autologous HSC are transduced *ex vivo* with gene vectors encoding supraphysiological levels of the lacking protein. Engraftment of these manipulated cells results in systemically elevated levels of the missing enzyme, thus pleiotropically correcting metabolism and organ function (Staal et al., 2019). Defective CNS metabolism is also restored because HSC and myeloid progenitors can cross the BBB (Fratantoni et al., 1968). Artificially enzyme-boosted microglia progenitors replace their ill counterparts in the brain, cross-correct neighboring cells and restore microglial function (Staal et al., 2019). Notably, the autologous nature of these HSC eliminates the possibility of graft rejection, GvHD and the need for a donor search. However, HSC-GT still requires a conditioning regimen to create a permissive bone marrow and brain niches. Therefore, treatment-related toxicity is not entirely abolished despite reduced intensity compared to Allo-HSCT.

In IEM with CNS involvement, correction of dysfunctional damage-response pathways is apparently enhanced with HSC that overexpress the missing enzymes. Therefore, enzyme-above-normal expression seems to represent the major driver of CNS demyelination arrest with HSC-GT, suggesting a “dose-dependent” treatment efficacy (Biffi, 2017). Through autologous use of HSC for enzyme deployment, this approach has brought brain targeting cellular drug vehicles into clinical practice. Varying degrees of experience regarding gene therapy in the IEM focused on this review currently exist.

MLD was one of the first LSD in which the efficacy of gene therapy was demonstrated. Recently, Fumagalli et al. (2022) published the long-term results of a phased 1/2 trial of lentiviral HSC-GT for patients with early-onset disease. Treatment was of meaningful clinical benefit, resulting in normal activity of the defective enzyme, which translated into prevention, stabilization, or reduced rate of neurological decline. Of note, enzyme activity in the cerebrospinal fluid of treated patients was shown to be restored to normal levels, suggesting efficient brain engraftment of enhanced cells (Fumagalli et al., 2022).

A phase 2–3 study of HSC-GT in 17 boys affected by CALD was published in 2017. Gene therapy led to positive outcomes in most patients with stable brain lesions and absent major functional disabilities in 15 out of the 17 treated boys (Eichler et al., 2017). Additional follow-up data to assess the durability of efficacy and long-term safety is still pending at the time of the writing of this review.

Recently, Gentner et al. (2021) published the interim results of a phase 1–2 study of HSC-GT in eight patients with Hurler Syndrome. After treatment, all patients acquired motor skills, and those presenting normal baseline function had no cognitive performance deterioration. Imaging revealed either stability or reduction of brain abnormalities. Additionally, all patients showed detectable enzyme and reduced substrate levels in the cerebrospinal fluid 3 months after treatment, which persisted until the latest follow-up (Gentner et al., 2021).

Compared to the other IEM presented in this review, HSC-GT is not yet used in KD because the supraphysiological expression of the affected lacking enzyme (galactocerebrosidade) in HSC is toxic. However, promising discoveries were reported in animal models by Gentner et al. (2010). The authors identified galactocerebrosidade-suppressing miRNAs specifically expressed in HSC that decrease during differentiation into mature hematopoietic cells. These findings support the investigation of HSC-GT in KD using these miRNAs, enabling successful gene therapy through mature hematopoietic-lineage cells while protecting HSC from galactocerebrosidade toxicity (Gentner et al., 2010).

HSC-GT represents a valuable addition to the therapeutic armamentarium for IEM with neurological involvement, thus far demonstrating encouraging results with rapid and profound CNS metabolic correction in affected patients.

Outside of the IEM setting, HSC-GT represents a developmental strategy for neurological disorders, currently being investigated in preclinical studies. HSC-derived tumor-infiltrating myeloid cells have been explored as a potential cellular drug vehicle in a mouse model of brain metastasis. Using promoters specifically upregulated in brain metastasis such as MMP14, Andreou et al. (2020) demonstrated that HSC-GT was effective in delivering pro-apoptotic molecules to the tumor, which resulted in prolonged survival of treated mice. Other neurological conditions such as Alzheimer’s disease, MS and Stroke are associated with brain-infiltrating myeloid cells that also overexpress MMP14 (Langenfurth et al., 2014), which, in the opinion of the authors of the study, creates a rationale for the use of HSC-GT in these benign neurological disorders (Andreou et al., 2020). In the future, HSC-GT application may expand as hematopoietic cells are increasingly being recognized to participate in the pathogenesis of several neurological conditions.

A summary of the several steps that take place with HSC-GT and the main advantages over Allo-HSCT in IEM are presented in Table 3.

**TABLE 3:** Steps of hematopoietic stem cell gene therapy and main advantages over Allo-HSCT in IEM with neurological involvement.

Steps of HSC-GT
1. Collection of autologous HSC
2. *Ex vivo* transduction of HSC with gene vectors encoding supraphysiological levels of the enzyme of interest
3. Busulfan-based conditioning regimen
4. Infusion of transduced HSC
5. Transduced HSC cross the BBB and engraft in the brain as microglia/macrophages
6. Microglial function is restored
7. Supraphysiological levels of the lacking enzyme are deployed into the brain (“Trojan Horse”)
**Main advantages over Allo-HSCT**
No need for a compatible donor
Shorter diagnosis-to-transplant time
Potential eligibility of patients with more advanced disease
Less intensive conditioning regimen
No need for GvHD prophylaxis
Absent risk of GvHD or graft rejection

*HSC-GT, hematopoietic stem cell gene therapy; HSC, hematopoietic stem cells; BBB, blood-brain barrier; Allo-HSCT, allogeneic hematopoietic stem cell transplantation; GvHD, graft vs. host disease.*

## Discussion

Certain neurological conditions are characterized by defects in cells that, despite residing in the central nervous system parenchyma, originate elsewhere. Immune-mediated Neurological Diseases and Inborn Errors Metabolism with CNS involvement are two paradigmatic settings of peripheral malfunctioning imported to the brain. In the former, peripheral immune cells that have exclusively lost self-tolerance to CNS components are the protagonists. In the latter, enzymatic substrate accumulation in the brain results in broad neural cell impairment that takes a particular toll on the myeloid-derived microglia.

Infusion of autologous HSC allows the delivery of immune-resetting high dose immunosuppression that would otherwise result in irreversible bone marrow failure. This approach has demonstrated meaningful clinical benefits in immune-mediated neurological diseases. In Multiple Sclerosis, a condition where phases of intense neuro-inflammation characterize clinical evolution, AHSCT can sucessfully eliminate autoreactive memory and redirect immune reconstitution to self-tolerance. Allo-HSCT brings additional benefits at the expense of increased toxicity, currently rendering its use developmental. Harnessing the graft-vs.-auto-immunity effect may represent a future avenue of investigation in immune-mediated neurological diseases.

Microglia dysfunction plays a central role in the pathogenesis of brain injury in Inborn Errors of Metabolism. The hematopoietic origin of microglia makes Allo-HSCT an efficient strategy for their replacement by donor-derived myeloid cells. Functional allogeneic microglia restore brain homeostasis by modulating inadequate responses to injury and cross-correcting neighboring metabolically impaired neural cells. After transplantation, changes in the cell composition and secretome of the myeloid brain compartment ultimately result in decreased substrate accumulation and enhanced repair mechanisms such as remyelination. Therefore, donor hematopoietic stem cells are integrated into the CNS cellular network of the recipient with profound therapeutic benefits.

Hematopoietic stem cell gene therapy explores HSC as a cellular enzyme vehicle to the brain. This treatment relies on the replacement of defective microglia by metabolically enhanced autologous HSC progeny that deliver supraphysiological levels of the deficient enzyme. Clinical benefits are encouraging and seem to depend on the above-normal enzyme expression. So far, vector-induced genotoxicity such as insertional mutagenesis has not been reported in this setting. Overall, this approach bypasses many of the limitations of Allo-HSCT and may potentially become standard treatment for these conditions. Importantly, long-term follow-up data will provide valuable information on the safety and persistence of metabolic correction. In the future, increased patient accrual to gene therapy clinical trials will bring to the surface the limitations and advantages of this approach over standard HSCT.

This review demonstrated that HSC represent a viable living therapy approach for certain devastating neurological disorders. Improved neurological outcomes and potential life-long reversal of brain injury mechanisms create a rationale for the use of this strategy in diseases such as IEM and immune-mediated neurological diseases that may be otherwise associated with a dismal short-term prognosis. However, given the serious complications that may arise from HSCT, careful patient selection is mandatory.

Experience of HSCT in Inborn Errors of Metabolism provides essential insights on the brain repairing mechanisms of both wild-type and modified hematopoietic stem cells. The central role of microglia in modulating maladaptive responses to injury and the feasibility of its replacement with current transplantation approaches provides a framework for future treatment pipelines in other demyelinating and neurodegenerative CNS conditions. Further investigation is needed to evaluate if the therapeutic potential of HSC can be successfully harnessed in different neurological disease settings.

## Author Contributions

PV wrote the manuscript. JL supervised the drafting and revision of the manuscript. Both authors contributed to the article and approved the submitted version.

## Conflict of Interest

The authors declare that the research was conducted in the absence of any commercial or financial relationships that could be construed as a potential conflict of interest.

## Publisher’s Note

All claims expressed in this article are solely those of the authors and do not necessarily represent those of their affiliated organizations, or those of the publisher, the editors and the reviewers. Any product that may be evaluated in this article, or claim that may be made by its manufacturer, is not guaranteed or endorsed by the publisher.

## Abbreviations

AHSCT: Autologous hematopoietic stem cell transplantation
ALD: X-Linked Adrenoleukodystrophy
Allo-HSCT: Allogeneic hematopoietic stem cell transplantation
BBB: Blood-brain barrier
CALD: Cerebral Adrenoleukodystrophy
CNS: Central nervous system
EBMT: European Group for Blood and Bone Marrow Transplantation
ERT: Enzyme replacement therapy
GvHD: Graft-vs.-host disease
HSC: Hematopoietic stem cells
HSCT: Hematopoietic stem cell transplantation
HSC-GT: Hematopoietic stem cell gene therapy
IEM: Inborn Errors of Metabolism
KD: Krabbe’s Disease
LSD: Lysosome Storage Diseases
MLD: Metachromatic Leukodystrophy
MPS IH: Mucopolysaccharidosis type I, Hurler variant
MS: Multiple Sclerosis
M6P: Mannose-6-phosphate.
